# Traumatic brain injury is associated with increased syndecan-1 shedding in severely injured patients

**DOI:** 10.1186/s13049-018-0565-3

**Published:** 2018-11-21

**Authors:** Erika Gonzalez Rodriguez, Jessica C. Cardenas, Charles S. Cox, Ryan S. Kitagawa, Jakob Stensballe, John B. Holcomb, Pär I. Johansson, Charles E. Wade

**Affiliations:** 1 Center for Translational Injury Research (CeTIR), Department of Surgery, McGovern Medical School, University of Texas Health Science Center, 6431 Fannin, MSB 5.204, Houston, TX 77030 USA; 2 Section for Transfusion Medicine, Capital Region Blood Bank, Copenhagen University Hospital, Rigshospitalet, Blegdamsvej 9, DK-2100 Copenhagen, Denmark; 3 Department of Anesthesia, Centre of Head and Orthopedics, Copenhagen University Hospital, Rigshospitalet, Copenhagen, Denmark; 4 Department of Pediatric Surgery, McGovern Medical School at The University of Texas Health Science Center, 6431 Fannin, MSB 5.258, Houston, TX 77030 USA; 5 Department of Neurosurgery, Mischer Neuroscience Institute, McGovern Medical School at The University of Texas Health Science Center, 6400 Fannin, Suite 2800, Houston, TX 77030 USA

**Keywords:** Endothelium, Syndecan-1, Traumatic endotheliopathy, Sympathoadrenal activation

## Abstract

**Introduction:**

Head injury and exsanguination are the leading causes of death in trauma patients. Hemorrhagic shock triggers systemic endothelial glycocalyx breakdown, potentially leading to traumatic endotheliopathy (EoT). Levels of syndecan-1, a main glycocalyx component, have been used to assess the integrity of the glycocalyx. In TBI patients, it remains unclear whether syndecan-1 shedding occurs and its correlation with outcomes. We aimed to determine the frequency of EoT+, defined as a syndecan-1 level of 40 ng/ml or higher, after TBI in isolated and polytraumatic injury. We also investigated how the presence of EoT+ affected outcomes in TBI patients.

**Methods:**

Severely injured trauma patients were enrolled. From blood samples collected upon patients’ arrival to the hospital, we measured syndecan-1 (main biomarker of EoT+), soluble thrombomodulin (sTM, endothelial activation) adrenaline and noradrenaline (sympathoadrenal activation), and assessed TBI patients’ coagulation capacity.

**Results:**

Of the enrolled patients (*n* = 331), those with TBI and polytrauma (*n* = 68) had the highest rate of EoT+ compared to isolated TBI (*n* = 58) and Non-TBI patients (*n* = 205) (Polytrauma-TBI 55.9% vs. Isolated-TBI 20.0% vs. non-TBI polytrauma 40.0%; *p = 0.001*). TBI patients with EoT+ exhibited marked increases in sTM, adrenaline and noradrenaline levels, and physiological and coagulation derangements. In isolated TBI patients, increasing syndecan-1 levels (β for every 10 ng/ml increase: 0.14; 95% CI: 0.02, 0.26) and hypocoagulability were negatively associated with survival.

**Conclusions:**

This study provides evidence of syndecan-1 shedding after TBI supporting the notion that breakdown of the glycocalyx contributes to the physiological derangements after TBI.

## Background

Head injury and hemorrhagic shock are the leading causes of death in trauma patients [1]. Most of these deaths occur in the first 24 h following injury, and it is during this time window that interventions that could potentially improve outcomes should be implemented [2–4]. While the implementation of strategies such as a balanced 1:1:1 plasma: platelets: red blood cell ratio has considerably reduced early mortality caused by hemorrhage, the proportion of early deaths attributed to acute traumatic brain injury (TBI) has remained unchanged [1]. Early interventions are limited partly because of a lack of understanding of the systemic pathogenesis of both isolated TBI and TBI with polytrauma [1, 4, 5].

Hemorrhagic shock leads to the systemic breakdown of the glycocalyx, a protective barrier on top of endothelial cells in blood vessels that is involved in the regulation of coagulation, inflammation, transcapillary flux, and mechanotransduction [6–8]. Alterations in these responses are believed to lead to traumatic endotheliopathy (EoT), a syndrome associated with high mortality [8–10]. Previous studies have demonstrated an association between the shedding of glycocalyx components and increased morbidity and mortality [11–13]. Using plasma samples collected from trauma patients, Rahbar et al. [11, 12] demonstrated that reductions in plasma oncotic pressure were associated with increased shedding of a major component of the glycocalyx, syndecan-1, and higher permeability (assessed in vitro). Patients with glycocalyx breakdown and lower plasma oncotic pressure were in shock, required a higher volume of blood transfusions, and had poorer survival than did patients without these characteristics [11, 12, 14]. Furthermore, a recent study from our laboratory demonstrated an association between acute low levels of serum albumin and increased glycocalyx breakdown in trauma patients [15]. The findings of these studies point to a state of increased permeability and endothelial dysfunction that leads to poorer outcomes [16–20].

The effects of shock and injury in the glycocalyx in patients with TBI are still not fully described, and whether the breakdown of the vascular glycocalyx can lead to EoT in TBI patients is unknown. In a porcine model of TBI and hemorrhage, Sillesen et al. [21] measured markers of endothelial activation and injury, inflammation, sympathoadrenal activation, and cell death. Syndecan-1 levels were significantly higher in animals subjected to TBI and hemorrhage than in controls. However, because the experimental group was exposed to both TBI and hemorrhage, whether the observed endotheliopathy resulted only from the systemic effects of hemorrhagic shock in the endothelium or also from the TBI-induced breakdown of the glycocalyx in cerebral blood vessels is unclear [21]. Further evidence suggested that the sympathoadrenal activation that occurs after head injury could drive the coagulopathic response seen in TBI patients [22, 23]. Di Battista and colleagues [23] also proposed a link between these responses and acutely increased levels of syndecan-1, as a biomarker of endotheliopathy, in isolated TBI patients with unfavorable outcomes.

Recently, we provided a quantitative definition of EoT and demonstrated that a cutoff syndecan-1 level of 40 ng/ml or higher (EoT+) identifies a group of patients who have an increased need for transfusion of blood products and higher mortality despite having similar admission physiology to patients without EoT [24]. However, the frequency of EoT, the factors associated with the development of this syndrome and its effects on outcomes in TBI patients are unknown. Here, we aimed to determine the frequency of EoT, defined as a syndecan-1 level of 40 ng/ml or higher, following TBI in the contexts of isolated and polytraumatic injury. We also investigated how the presence of glycocalyx breakdown, as measured by syndecan-1, affected outcomes in TBI patients. We hypothesized that head injury can lead to EoT and that the presence of severe TBI in combination with polytrauma potentiates glycocalyx breakdown as evidenced by syndecan-1 shedding. We expected that patients with both TBI and polytrauma would more frequently have EoT+ than would patients with either TBI alone or polytrauma alone. Identifying patients at risk of EoT paves the way for the development of clinical studies that investigate how best to treat patients in need of endothelial repair. Furthermore, this study contributes to the understanding of EoT and its effects on the clinical outcomes of TBI patients.

## Methods

### Study design and sample analysis

For this prospective observational study, we obtained approval from The University of Texas Health Science Center at Houston Institutional Review Board (HSC-GEN-12-0059). This study included previously reported data from 247 patients [8, 24, 25]. Adult patients who were admitted to our level 1 trauma center from July 2011 to May 2016 and who required trauma team activation were eligible for inclusion. Patients were excluded if they were pregnant, were prisoners, were enrolled in other studies, declined to consent, or if no blood sample was drawn on admission. Consent was obtained from the patient or a legally authorized representative within 72 h of admission or waived for patients who were discharged or died within 24 h of hospital admission. No changes in clinical practice were implemented in this observational study.

We collected 20 ml of blood in citrated tubes upon patients’ arrival to the emergency department. Blood was transferred into vacutainer tubes containing 3.2% citrate and inverted to ensure proper anticoagulation. Rapid thrombelastography (rTEG) were performed according to the manufacturer’s instructions. Samples were then centrifuged at 3200 rpm for 20 min at room temperature. After spinning, plasma was aliquoted and frozen for later analysis. In addition, we collected blood samples from healthy female and male volunteers to serve as controls.

**Fig. 1:**
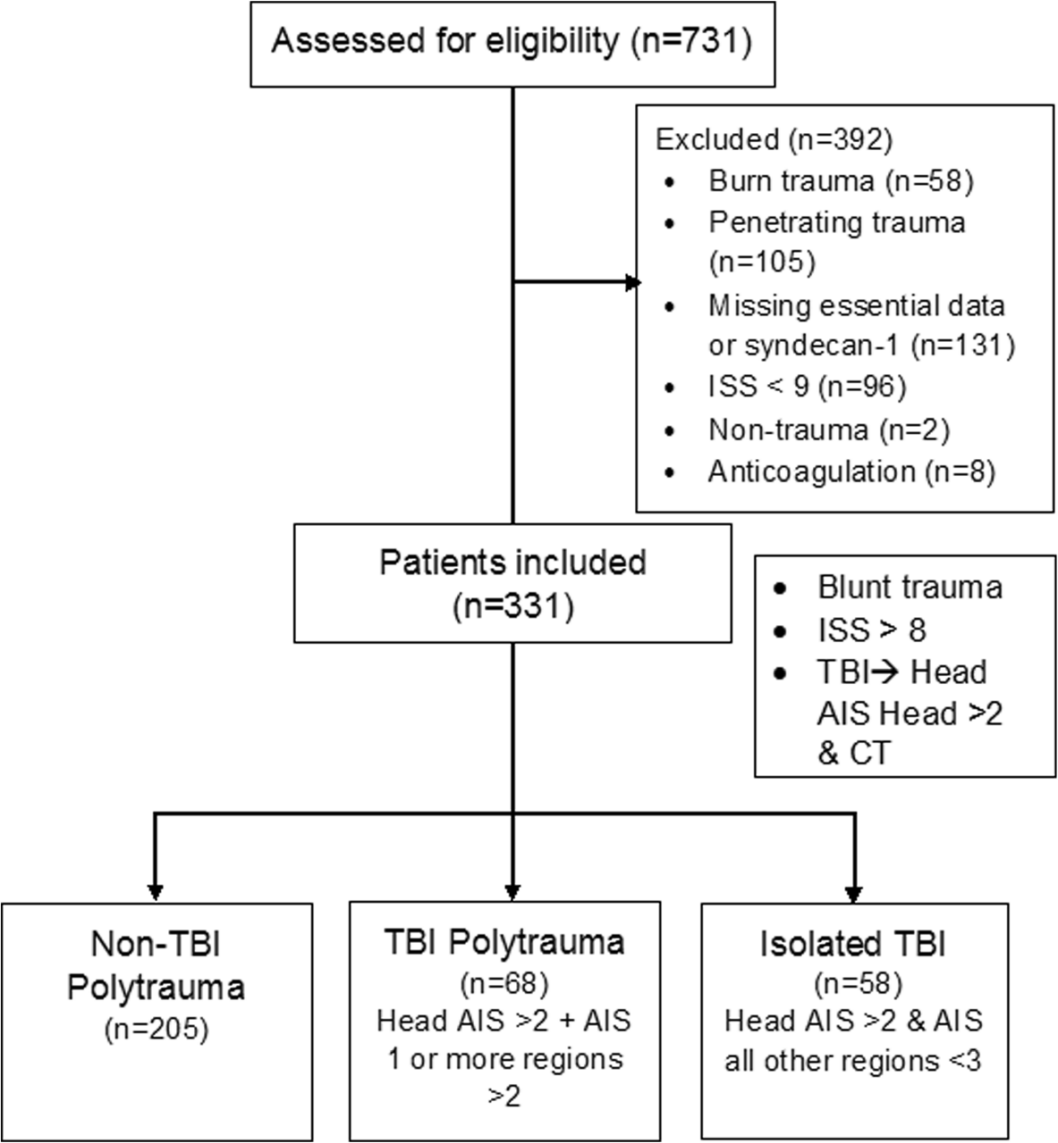
Study flow diagram showing the selection process used to identify trauma patients for inclusion in the study. ISS, Injury Severity Score; TBI, traumatic brain injury; AIS, Abbreviated Injury Scale; CT, computed tomography

We only included patients with blunt trauma and an injury severity score (ISS) above 8 for whom syndecan-1 levels and other essential data were available. Syndecan-1 levels were also measured from 29 healthy volunteers. Of the 731 patients assessed for eligibility, 41 patients were excluded because of missing syndecan-1 level data, 96 patients were excluded because they had an ISS of less than 9, and 105 patients were excluded because they had penetrating trauma. Out of the excluded patients with penetrating injury, 11 had TBI. All of these patients sustained isolated injuries to the head (82.0% gunshot wounds), and three subsequently died. Eight more patients in the TBI group were excluded because they were on anticoagulation at the time of injury (Fig. 1). The demographic profiles of the excluded patients with mild trauma (ISS < 9) were similar to those of patients included in the study and were representative of our trauma patient population (age: median 35 years, interquartile range [IQR] 27–53 years; sex: 77% male; mortality rate: 10.4%). By comparing the study cohort to the overall trauma patient population, we ensured that we were not excluding any particular demographic group from the analysis, except for patients that suffered penetrating trauma. Furthermore, data from 240 patients included in this study have been previously reported by Johansson and colleagues [8, 25], and Gonzalez Rodriguez et al.

Patients were classified as having severe TBI if their Abbreviated Injury Scale (AIS) Head/Neck score was higher than 2, and there was radiological confirmation of TBI with computed tomography (CT). The attending trauma surgeon judges the need of obtaining an initial head CT scan based on arrival Glasgow Coma Score (GCS), mechanism of injury and clinical criteria. Subsequently, both a radiologist and the trauma team interpret the head CTs and request consultations from neurosurgery if needed. Patients with an AIS Head/Neck score higher than 2 and an AIS score in all other regions lower than 3 were classified as having isolated TBI. Patients with TBI and an AIS score in at least one region of more than 2 were classified as polytrauma-TBI. Patients without TBI, but with an AIS score in at least two regions higher than 2 were defined as non-TBI polytrauma. The types of brain lesion were assessed and classified into 5 mutually exclusive categories: subarachnoid hemorrhage, subdural hemorrhage, epidural hematoma, intraparenchymal contusion, and multifocal intracranial hemorrhage (MIH; in cases where 2 or more brain lesions were observed, but neither was dominant).

Designated study personnel prospectively collected patient demographics, vital signs, standard laboratory values, transfusions data, arrival GCS, and information on the mechanisms and severity of injuries at the time of admission. Outcomes, including hospital and intensive care unit-free days and 30-day in-hospital mortality, were obtained from medical records.

### Enzyme-linked immunosorbent assays

Our main biomarker of interest, syndecan-1, was quantified from plasma obtained at hospital arrival using a commercially available enzyme-linked immunosorbent assay (Diaclone SAS, Besancon, France; lower limit detection 4.94 ng/ml). We also measured: soluble thrombomodulin, a marker of endothelial cell activation or dysfunction, was quantified using a commercially available enzyme-linked immunosorbent assay (sTM Nordic Biosite, Copenhagen, Denmark; lower limit detection 0.31 ng/ml) according to manufacturer recommendations. Adrenaline (2-CAT ELISA, Labor Diagnostica Nord GmbH & Co. KG, Nordhorn, Germany; lower limit of detection 10 ρg/ml; normal reference level < 100 ρg/ml) and noradrenaline (2-CAT ELISA, Labor Diagnostica Nord GmbH & Co. KG, Nordhorn, Germany; lower limit of detection 50 ρg/ml; normal reference level < 600 ρg/ml) as indicators of sympathoadrenal activation and drivers of EoT [8, 25].

### Coagulation assessment by viscoelastic hemostatic assays

Rapid thrombelastography tests (rTEGs) were run on a Thrombelastograph 5000 (Hemoscope Corp, Niles, IL) by technicians at the Memorial Hermann Hospital emergency department, according to manufacturer’s recommendations. The activated clotting time (ACT) was defined as the time from the start of the test to fibrin formation. Values equal to or more than 128 s are highly predictive of the need for massive transfusion within 6 h [26]. The maximum amplitude (MA) is indicative of the contribution of platelet function and platelet-fibrin interactions to clot strength (reference value: 54–72 mm). LY30 is the percent amplitude reduction at 30 min after MA (reference value 0.0–7.5%).

### Statistical analysis

Sample size calculation was performed to detect a difference of syndecan-1 levels of at least 12 ng/ml between healthy volunteers and trauma patients’ mean levels. We determined that at least 27 patients per group were needed. Based on our previous work, we used a cutoff syndecan-1 level of ≥40 ng/ml to define EoT+ [area under the curve 0.70, 95%CI 0.58–0.84) [24]. Mann-Whitney U or Kruskal-Wallis tests were used for comparisons between and across groups. Post-hoc analyses were performed with Dunn’s tests. The chi-square test was used for comparisons between categorical variables. Summary statistics (medians with interquartile ranges (IQR)) were used to describe continuous variables, and categorical data were presented as frequencies and percentages. With multivariable logistic regression, we determined factors associated with the likelihood of 30-day mortality in patients with isolated TBI. A purposeful selection of covariates approach was followed to fit the model. Initially, we considered variables known to be clinically significant in trauma and TBI that were collected within 3 h of hospital arrival (demographic characteristics, vital signs, arrival physiology, and mechanism of injury). From the univariate analysis, we selected for inclusion in the preliminary effects model those variables that were significant at the *p* < 0.2 level. Checks for interactions were made, and goodness of fit of the model was assessed with the Hosmer-Lemeshow goodness-of-fit test. Statistical significance was set at the *p* < 0.05 level. All statistical analyses were performed with Stata software version 13.0 (StataCorp, College Station, TX).

## Results

### Patient characteristics

Of 581 patients with plasma samples available, 331 met the inclusion criteria. Forty percent of them (*n* = 126) had TBI (polytrauma, *n* = 68; isolated TBI, *n* = 58). Most patients in the complete cohort were moderately injured (ISS: median 22, IQR 16–29) males (71.3%). These patients typically had altered mental status on arrival (GCS: median 5, IQR 3–15) but tended to be normotensive (systolic blood pressure: median 119 mmHg, IQR 99–140) and to have normal arrival hemoglobin levels (median 13.0, IQR 11.6–14.2 g/dl) and platelet counts (median 220, IQR 183–270). The rate of EoT+ (syndecan-1 level ≥ 40 ng/ml) was 39.8%, similar to the rate in our previous report [24]. Survival at 30 days was 79.5% (Table 1). In terms of type of brain lesion, 37.3% (*n* = 47) of patients presented with subdural hemorrhage, 23.0% (*n* = 29) with subarachnoid hemorrhage, 21.4% (*n* = 27) with MIH, 7.1% (*n* = 9) with epidural hematoma and 11.1% (*n* = 14) with intraparenchymal hemorrhage.

**Table 1:** Demographic and clinical characteristics of 331 trauma patients

	All patients *N* = 331	Non-TBI Polytrauma *N* = 205	Poly TBI *N* = 68	Isolated TBI *N* = 58	*P* value
Demography					
Age, y	43 [29–58]^a^	42 [27–57]	44 [34–58]	50 [33–76]	0.12
Male, *n* (%)	236 (71.3)	145 (70.7)	52 (76.5)	39 (67.2)	0.5
ISS	22 [16–29]	19 [14–27]	34 [27–41]^d^	25 [16–26]^e^	< 0.0001
Head AIS = 3, *n* (%)	45 (35.7)^b^	–	30 (44.1)	15 (25.9)	0.015
Head AIS = 4, *n* (%)	35 (27.8)^b^	–	21 (30.9)	14 (24.1)	
Head AIS ≥ 5,* n* (%)	45 (35.7)^b^	–	16 (23.5)	29 (50.0)	
ED Vital Signs and Laboratory Values					
SBP, mmHg	119 [99–140]	114 [93–137]^f^	116 [100–139]	140 [128–158]^e^	0.0001
HR, beats/min	95 [79–112]	96 [83–119]^f^	93 [77–111]	86 [70–102]	0.004
BE, mEq/l	−4 [−7 to 0]	− 4 [− 7 to − 1]^f^	−5 [− 8 to − 2]	− 1 [− 5 to 2]^e^	0.0001
Platelet count, 10^9^/l	221 [185–270]	224 [189–278]^f^	222 [189–264]	206 [172–256]	0.2
Hemoglobin, g/dl	13.0 [11.6–14.2]	13.0 [11.6–14.4]	13.2 [11.5–13.9]	13 [11.9–14.4]	0.86
GCS	6 [3–15]	10 [3–15]^f^	3 [3–9]^d^	3 [3–9]	0.0001
Biomarkers					
sTM, ng/ml	6.2 [4.6–8.4]	6.1 [4.4–8.2]	6.5 [5.1–8.8]	5.6 [4.3–9.3]	0.24
Syndecan-1, ng/ml	33.2 [16.6–79.1]	31.5 [15.5–61.2]	54.0 [25.1–107.0]^d^	25.0 [15.5–44.4]^e^	0.0007
EoT+, *n* (%)	132 (39.8)	82 (40.0)	38 (55.9)^d^	12 (20.6)^e^	0.001
Outcomes					
Transfused, *n* (%)	191 (58.1)	114 (55.6)	50 (73.5)^d^	27 (46.5)^e^	0.014
24-h mortality, *n* (%)	18 (5.4)	7 (3.4)^f^	4 (5.9)	6 (10.3)	0.025
48-h mortality, *n* (%)	37 (11.2)	16 (7.8)^f^	9 (13.2)	12 (20.7)	0.02
72-h mortality, *n* (%)	40 (12.1)	16 (7.8)^f^	11 (16.2)^d^	13 (22.4)	< 0.0001
30-day mortality, *n* (%)	68 (20.5)	27 (13.2)^f^	19 (27.9)^d^	22 (37.9)	< 0.0001

*Abbreviations*: *TBI* traumatic brain injury, *ISS* Injury Severity Score, *AIS* Abbreviated Injury Score, *ED* emergency room, *SBP* systolic blood pressure, *HR* heart rate, *BE* base excess, *GCS* Glasgow Coma Scale, *sTM* soluble thrombomodulin, *EoT* traumatic endotheliopathy

^a^Values are medians and interquartile ranges (IQR) unless otherwise specified

^b^Only in 126 patients

^c^Data for only 226 patients

^d^Statistically significant difference between the Polytrauma TBI and Non-TBI polytrauma groups

^e^Statistically significant difference between the Polytrauma TBI and Isolated TBI groups

^f^Statistically significant difference between the Non-TBI and Isolated TBI groups

### Syndecan-1 shedding in patients with isolated TBI, polytrauma in combination with TBI, and polytrauma only

**Fig. 2:**
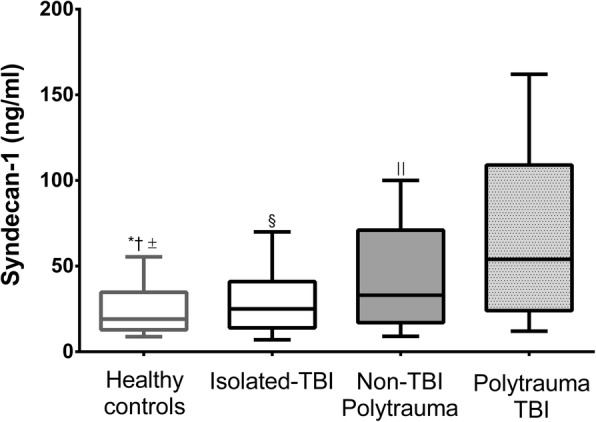
Admission syndecan-1 levels in healthy volunteers (*n* = 29), patients with isolated TBI (*n* = 58), TBI combined with polytrauma (*n* = 68) and polytrauma only (*n* = 205). * Statistically significant difference between Isolated TBI patients vs. Healthy controls. † Statistically significant difference between Healthy controls vs. Non-TBI polytrauma patients. ± Statistically significant difference between Healthy controls vs. Polytrauma TBI patients. § Statistically significant difference between Isolated TBI patients vs. Polytrauma TBI patients. || Statistically significant difference between Polytrauma TBI patients vs. Non-TBI polytrauma patients.

In order to determine whether TBI in the presence of polytrauma exacerbated the breakdown of the glycocalyx and resulted in a higher frequency of EoT+, we assessed and compared levels of our biomarker of interest (syndecan-1) across patients with isolated TBI, polytrauma and TBI, and polytrauma without TBI. Syndecan-1 levels in controls were significantly lower than in non-TBI polytrauma (median: 19.1 ng/ml vs. 31.5 ng/ml; *p =* 0.009*)*, isolated TBI (median: 19.1 ng/ml vs. 25.0 ng/ml; *p* < 0.05) and polytrauma-TBI patients (median: 19.1 ng/ml vs. 54.0 ng/ml; *p =* 0.0001). We observed significant increases in shedding of syndecan-1 in polytrauma-TBI patients compared to those with polytrauma but no TBI (non-TBI polytrauma) (*p* = 0.001) and those with isolated TBI (*p* < 0.001). Median syndecan-1 levels in the polytrauma-TBI group were 1.5 times higher than in the non-TBI polytrauma group (54.0 ng/ml vs. 31.5 ng/ml) and more than 2 times higher than those in the isolated TBI group (54.0 ng/ml vs. 25.2 ng/ml) (Fig. 2). Conversely, sTM levels were comparable across groups, indicating similar levels of endothelial activation or dysfunction. The majority of polytrauma-TBI patients were EoT+ (*n* = 38, 55.9%); in the other two groups, this proportion was significantly lower (*p* = 0.001) (Table 1).

### Subgroup comparisons between EoT+ and EoT− patients with TBI

We stratified TBI patients according to the presence (EoT+) or absence (EoT−) of EoT. EoT+ patients (*n* = 50, 39.7%) had higher ISS, base deficit, and were more likely to be tachycardic, hypotensive, and coagulopathic on arrival than were EoT− patients. Furthermore, EoT+ patients had higher levels of biomarkers indicative of sympathoadrenal activation (adrenaline and noradrenaline) and endothelial activation/dysfunction (sTM).

EoT+ patients more frequently required in-hospital transfusion of blood products. There was also a trend towards fewer hospital-free days, but it was not significant (*p* = 0.06). The mortality rate was similar between groups (Table 2). The frequency of complications was higher in patients with EoT+. For example, all patients in our cohort that had acute kidney injury (*n* = 11; *p* < 0.0001) and acute respiratory distress syndrome (*n* = 4; *p* = 0.023) had EoT+. When accounting for the frequency of all complications associated with endotheliopathy (pulmonary embolism, deep vein thrombosis, sepsis, acute respiratory distress syndrome (ARDS) and acute kidney injury), the proportion of EoT+ patients with one or more complications was four times higher than that of the EoT- group (*n* = 19, 38.0% vs 9.2%, *n* = 7; *p* = 0.003).

**Table 2:** Clinical characteristics and outcomes comparing 126 TBI patients (isolated TBI and TBI and polytrauma) with EoT+ (syndecan-1 ≥ 40 ng/ml) vs. EoT- (syndecan-1 < 40 ng/ml) and 205 polytrauma patients without TBI

	Polytrauma only		*P value*	TBI		*P value*
	EoT- (*N* = 123)	EoT+ (*N* = 82)		EoT- (*N* = 76)	EoT+ (*N* = 50)	
ISS	17 [13–26]	24 [17–30]	0.001	25 [18–29]	32 [26–41]	< 0.001
SBP, mmHg	103 [88–126]	120 [98–142]	0.001	135 [116–150]	115 [100–140]	0.006
HR, beats/minute	96 [84–114]	97 [81–120]	0.9	84 [70–104]	100 [84–111]	0.007
BE, mEq/l	−3 [−6 to 0]	−6 [−8 to −2]	0.002	− 3 [−5 to 0]	−5 [− 8 to − 1]	0.01
GCS	13 [3–15]	7 [3–15]	0.34	3 [3–9]	3 [3–7]	0.7
Polytrauma, n (%)	–	–		31 (39.2)	39 (70.9)	< 0.0001
rTEG values						
ACT, seconds	113 [105–121]	121 [113–125]	0.04	105 [105–121]	121 [105–136]	0.004
MA, mm	65 [62–69]	63 [58–68]	0.03	65 [60–69]	62 [58–67]	0.006
α- angle, degrees	75 [72–78]	73 [70–77]	0.004	73 [70–77]	72 [69–76]	0.12
Biomarkers						
sTM, ng/ml	5.0 [3.8–6.9]	7.9 [5.6–9.4]	< 0.001	5.1 [4.3–7.2]	7.4 [5.8–9.9]	< 0.0001
Syndecan-1, ng/ml	19.3 [11.3–30.2]	94.4 [59.0–155.6]	N/A	17.9 [11.6–30.5]	109.6 [71.1–217.8]	N/A
Adrenaline^a^, ρg/ml	93.4 [36.7–287.6]	185.7 [64.4–618.8]	0.01	106.7 [38.0–311.4]	339.0 [132.6–786.5]	< 0.001
Noradrenaline^a^, ρg/ml	846.2 [312.9–1340.3]	1530.2 [570.1–1825.9]	0.03	588.9 [345.9–1148.9]	1143.1 [589.2–1866.1]	< 0.001
Outcomes						
Transfused, n (%)	49 (39.8)	65 (79.3)	< 0.01	36 (47.4)	37 (74.0)	0.003
Hospital-free days	18 [6–25]	12 [0–21]	0.03	10 [0–20]	2 [0–12]	0.06
ICU-free days	27 [20–30]	23 [11–29]	0.01	17 [0–27]	12 [0–22]	0.14
24-h mortality, n (%)	1 (0.8)	6 (7.3)	0.02	5 (6.6)	5 (10.0)	0.5
48-h mortality, n (%)	4 (3.2)	9 (11.0)	0.04	12 (22.4)	9 (18.0)	0.8
72-h mortality, n (%)	6 (4.9)	10 (12.2)	0.07	12 (22.4)	12 (24.0)	0.25
30-day mortality, n (%)	13 (10.6)	14 (17.1)	0.1	24 (31.5)	17 (34.0)	0.6

*Abbreviations*: *TBI* traumatic brain injury, *EoT* traumatic endotheliopathy, *ISS* Injury Severity Score, *AIS* Abbreviated Injury Score, *SBP* systolic blood pressure, *HR* heart rate, *BE* base excess, *GCS* Glasgow Coma Scale, *rTEG* rapid thrombelastography, *ACT* activated clotting time, *MA* maximum amplitude, *sTM* soluble thrombomodulin, *ICU* intensive care unit

^a^Data available for only 226 patients

### Syndecan-1 shedding in isolated TBI

**Fig. 3:**
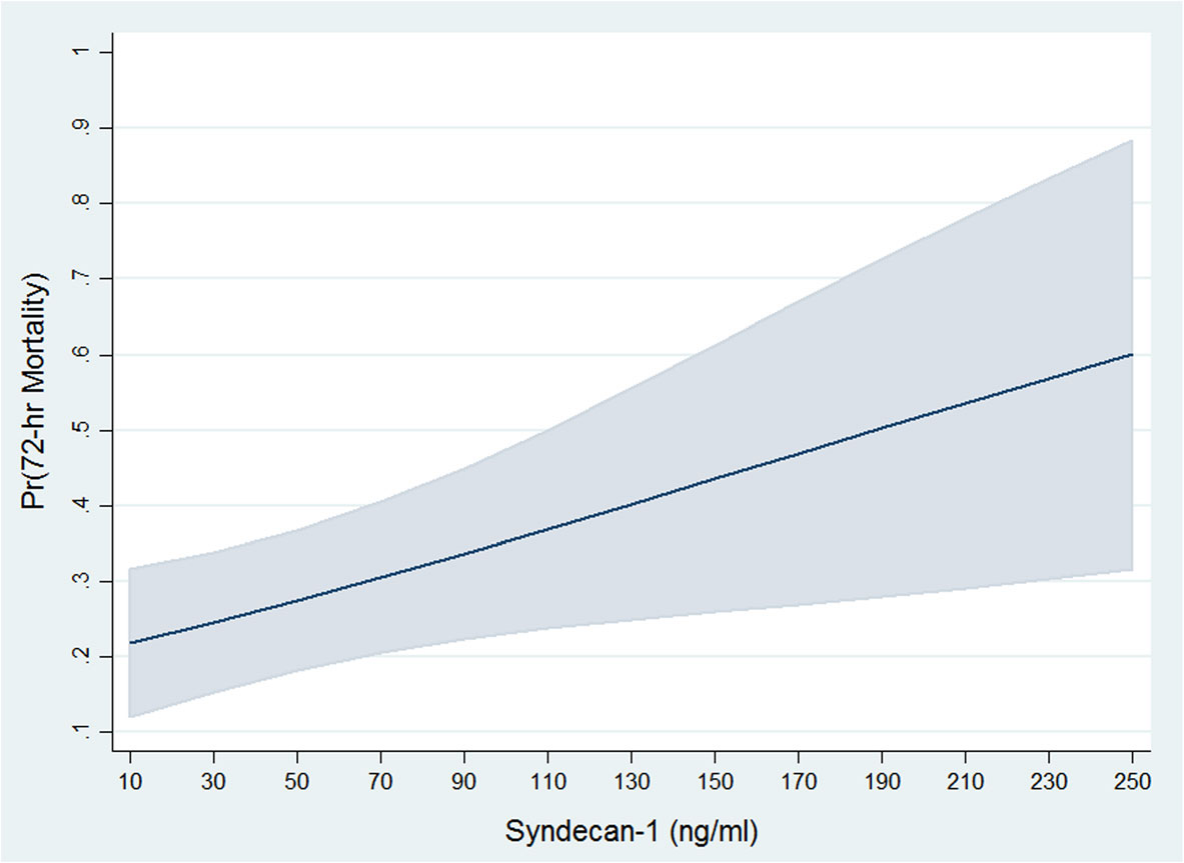
Marginal plot of the effect of ED syndecan-1 level on the predicted probability of 72-h mortality^*^ in 58 patients with isolated TBI ^*Shaded region indicates 95% confidence intervals^

Because we wanted to determine how the breakdown of the glycocalyx contributed to poor outcome in the context of TBI, we performed a subgroup analysis including patients with isolated TBI only. Following a purposeful selection of variables approach to covariate selection, we fitted a multiple logistic regression to assess whether increasing shedding of syndecan-1 was associated with higher 72-h mortality. We chose deaths within 72 h of hospital arrival as the majority of deaths in our trauma center occur within this period [1]. We initially considered demographic data, injury severity indices, vital signs and syndecan-1 as variables that could be clinically meaningful, but only included in the preliminary effects model those variables with a *p*-value of less than 0.25. The variables that met these criteria were age, arrival systolic blood pressure, syndecan-1, rTEG MA and ISS. After covariate selection and fitting the final effects model, we found that along with older age, higher ISS and lower rTEG MA, increasing syndecan-1 levels were independently associated with a higher likelihood of dying at 30 days (Table 3). We checked for potential interactions between syndecan-1 levels and ISS, and syndecan-1 levels and rTEG MA. None of the interactions were statistically significant or influenced importantly the effect of the other variable in the interaction term, thus they were not included in the final effects model. The Hosmer-Lemeshow test for goodness-of-fit of the model was not statistically significant, indicating a good fit of the model (goodness-of-fit test *p* = 0.8). Based on this model, we evaluated the marginal effects at different levels of syndecan-1 on the predicted probability of death at 72 h while all other covariates were left at their observed values. Our estimation suggests that, at least in this sample, the predicted probability of 72-h mortality rises with syndecan-1 levels in patients with isolated TBI and is about 26% [95% CI 17–35.2%] for patients with syndecan-1 levels of 40 ng/ml. For levels of equals of more than 100 ng/ml (13.8% of patients had levels of or above this threshold), the predicted probability is at least 35.3% (Fig. 3).

**Table 3:** Univariate and multivariable logistic regression analyses of factors associated with 30-day in-hospital mortality in 58 patients with isolated traumatic brain injury

	Univariate (n = 58)		Multivariate (n = 58)	
	β (95% CI)	*p-value*	β (95% CI)	*p-value*
Syndecan-1 (10 ng/ml)	0.04 (-0.02 – 0.10)	0.1	0.14 (0.02 - 0.26)	0.02
Age (yr)	0.05 (0.02 – 0.07)	0.001	0.06 (0.02 - 0.1)	0.005
ISS	0.2 (0.09 – 0.32)	0.001	0.29 (0.1 - 0.46)	0.001
rTEG MA (mm)	-0.8 (-0.14 to -0.01)	0.02	-0.15 (-0.2 to -0.003)	0.04
Systolic blood pressure (mmHg)	-0.12 (-0.3 – 0.01)	0.18	-0.02 (-0.05 - 0.01)	0.3
Interactions				
Syndecan-1 x ISS	NA	NA	0.002 (-0.48 – 0.08)	0.72
Syndecan-1 x rTEG MA	NA	NA	-0.01 (-0.05 – 0.03)	0.59

Regression coefficients (β) with 95%confidence intervals (95%CI) and *p* values are displayed

*Abbreviations*: *ISS* Injury severity score, *rTEG MA* rapid thrombelastography maximum amplitude, *NA* not applicable

## Discussion

The current study provides evidence of the presence of EoT after TBI and supports the notion that breakdown of the glycocalyx is a contributor to the physiological derangements after TBI. In a cohort of severely injured patients, we found that in patients with isolated TBI, rising syndecan-1 levels were an independent factor associated with higher odds of 72-h mortality. Furthermore, we found that polytrauma in the presence of TBI exacerbated the breakdown of the glycocalyx, leading to increased circulating levels of syndecan-1 and a higher frequency of EoT (defined as syndecan-1 ≥ 40 ng/ml) in these patients compared to those with isolated TBI or polytrauma only.

Polytrauma in combination with TBI potentiated glycocalyx breakdown, resulting in a higher frequency of EoT+ in these patients. This could have arisen from the activation of the sympathetic nervous system, which seems to drive endothelial dysfunction, glycocalyx breakdown, and coagulopathy both in severely injured trauma patients and in those with isolated TBI [22, 23]. Interestingly, sTM levels were comparable across the three groups (non-TBI polytrauma, polytrauma TBI and isolated TBI) indicating a similar degree of endothelial activation/dysfunction.

TBI patients with syndecan-1 ≥ 40 ng/ml (EoT+) had physiological derangements shortly after injury and were more likely to have lower arrival systolic blood pressure, higher heart rate and base deficit, altered rTEG parameters compatible with coagulopathy (lower MA and prolonged ACT) and increased sympathoadrenal activation. Both hemodynamic instability and coagulopathy are components of EoT [8, 25]. sTM is a marker of endothelial activation or dysfunction and it is expected to increase with higher glycocalyx breakdown (i.e. syndecan-1 shedding) [8, 25]. The fact that TBI patients with EoT+ had significantly higher levels of adrenaline and sTM is consistent with the current notion of how endotheliopathy ensues, where the exacerbated sympathoadrenal activation following injury triggers glycocalyx breakdown, which leads to endothelial dysfunction and ultimately to endotheliopathy [8]. Our findings are consistent with what was reported by Di Battista and colleagues in a study conducted in patients with blunt isolated TBI. In this study, the authors found that in patients with blunt isolated TBI, the levels of biomarkers of sympathoadrenal activation (adrenaline and noradrenaline) co-varied with biomarkers of endotheliopathy including syndecan-1.

The breakdown of the glycocalyx exposes endothelial cells to circulating cytokines, immune cells, and other factors that trigger several responses, potentially leading to EoT [10, 12, 13, 21, 27]. Until recently, most clinical studies have only evaluated the integrity of the endothelium and glycocalyx in the context of trauma-induced coagulopathy after TBI [23, 28]. However, since the glycocalyx is involved in a series of physiological responses, its breakdown and associated EoT can have far greater implications [6, 18]. For example, we can speculate that the glycocalyx plays a role in the regulation of the blood-brain barrier’s permeability and that glycocalyx breakdown may contribute to the development of cerebral edema or to hemorrhagic progression. In a model of combined TBI and hemorrhagic shock, Halaweish et al. [29, 30] demonstrated that early treatment with fresh frozen plasma reduces the size of the brain lesion, attenuates endothelial activation, and improves neurological recovery. These positive effects of plasma could be to some extent attributable to the attenuation of the EoT through the repair of the glycocalyx [18, 31, 32].

One of the main goals of this study was to determine whether the breakdown of the glycocalyx was associated with poor outcomes in patients with TBI. We found here that after adjusting for potential confounders, increasing syndecan-1 levels were an independent factor associated with higher odds of 72-h mortality in patients with isolated TBI. In addition, we did not find any significant interactions between syndecan-1 levels and ISS score, indicating that in these patients, the increased shedding of the glycocalyx is not merely a direct result of the severity of anatomical injury. These findings suggest that even though patients with isolated TBI seem to have less severe EoT, the breakdown of the glycocalyx may be significant enough to be a contributing factor to poor outcomes. When comparing TBI patients with and without EoT, we could only observe a trend towards fewer hospital-free days in patients with TBI and EoT+, but this trend was not statistically significant. In addition, we could not observe any differences in the frequency of mortality between these two groups. Patients in both groups were severely injured (ISS > 25) irrespective of EoT status, which could have limited our capacity to observe small differences in these parameters in a relatively small cohort of patients. One of the components of the EoT is tissue injury and organ dysfunction [9]. Several studies have reported that sepsis, acute kidney injury and thrombosis are in part the result of endothelial dysfunction and glycocalyx breakdown [25]. The lung is one of the organs with the highest surface of glycocalyx. Pulmonary inflammation and vascular leakage mediated by glycocalyx breakdown are hallmarks of ARDS [33]. Although the frequency of ARDS is very low in our trauma center due to the limited use of crystalloids, all ARDS cases in this cohort had syndecan-1 levels > 40 ng/ml. EoT+ patients with TBI were significantly more likely to have at least one of the aforementioned complications. The manifestation of these complications along with an exacerbated sympathoadrenal surge, increased glycocalyx breakdown, endothelial dysfunction and coagulopathy suggests that the traumatic endotheliopathy, as a systemic “disease”, also occurs following TBI.

This study has several limitations. We included only patients with blunt trauma and moderate to severe ISS with radiologically confirmed TBI; however, by limiting our cohort to these patients, we were ensuring that we were studying patients who could benefit from potential early interventions that target the glycocalyx. Another limitation comes from employing head AIS and head CT scan to identify patients that had severe TBI. As opposed to head AIS, GCS scoring can be employed in the field to identify patients with altered mental status, and it is widely used to assess head injury severity. However, about half of the patients classified as Non-TBI polytrauma arrived with altered mental status (arrival GCS below thirteen) and had no confirmation of TBI. This is likely due to interventions on the field, for example, intubation, or other causes such as intoxication. Subsequently, we decided to use head AIS and CT confirmation to identify those patients that suffered from severe TBI. Mortality rates in TBI patients could be confounded by the withdrawal of life-sustaining therapies, which could predispose older patients to have life-sustaining therapies withdrawn at an earlier stage because of a perceived poor prognosis [34]. Turgeon and colleagues found that most deaths in TBI patients occurred after the withdrawal of life-sustaining therapies [35], a limitation shared by several studies with human TBI patients. Nonetheless, after controlling for age (and other factors), we found that increasing levels of syndecan-1 were an independent factor of mortality in patients with isolated TBI. We evaluated circulating syndecan-1 as the defining biomarker of EoT, but several other unknown mechanisms are involved in this syndrome. Because the biomarker levels measured correspond to circulating levels that result from breakdown of the endothelial glycocalyx of blood vessels systemically, we cannot know how extensive the breakdown of the glycocalyx of cerebral blood vessels is. Nevertheless, previous studies from our group as well as substantial animal and clinical evidence support the role of circulating syndecan-1 in EoT and its associations with poor outcomes in trauma patients. The relatively small sample size in the isolated and polytrauma TBI groups is also a limitation. Nevertheless, we were able to detect statistically significant differences in levels of syndecan-1 across the groups [8, 12, 23–25, 27].

## Conclusions

In conclusion, TBI combined with polytrauma markedly exacerbates glycocalyx breakdown, leading to a higher frequency of EoT+ than does isolated TBI or polytrauma without TBI. TBI patients with EoT+ (syndecan-1 ≥ 40 ng/ml) exhibit marked endothelial dysfunction, physiological and coagulation derangements, and a higher frequency of complications. Even though patients with isolated TBI seem to experience less glycocalyx breakdown, as measured by circulating levels of syndecan-1, after adjusting for several factors including injury severity, increasing levels of this biomarker were an independent factor associated with higher 72-h mortality in this population.

## Abbreviations

AIS: Abbreviated Injury Scale
ARDS: Acute respiratory distress syndrome
CT: Computed tomography
ED: Emergency room
EoT: Traumatic endotheliopathy
GCS: Glasgow coma score
IQR: Interquartile range
ISS: Injury severity score
MIH: Multiple intracranial hemorrhage
rTEG: Rapid thrombelastography
sTM: Soluble thrombomodulin
TBI: Traumatic brain injury
